# Comparison and Evaluation of Gridded Precipitation Datasets in a Kansas Agricultural Watershed Using SWAT

**DOI:** 10.1111/1752-1688.12819

**Published:** 2020-05-16

**Authors:** Muluken E. Muche, Sumathy Sinnathamby, Rajbir Parmar, Christopher D. Knightes, John M. Johnston, Kurt Wolfe, S. Thomas Purucker, Michael J. Cyterski, Deron Smith

**Affiliations:** Office of Research and Development, U.S. Environmental Protection Agency, Athens, Georgia, USA;; Oak Ridge Institute for Science and Education (ORISE) Postdoctoral Research Participant at Office of Research and Development, U.S. Environmental Protection Agency, Athens, Georgia, USA;; Office of Research and Development, U.S. Environmental Protection Agency, Athens, Georgia, USA;; Office of Research and Development, U.S. Environmental Protection Agency, Narragansett, Rhode Island, USA; Independent Contractor at Office of Research and Development, U.S. Environmental Protection Agency, Athens, Georgia, USA; Office of Research and Development, U.S. Environmental Protection Agency, Athens, Georgia, USA;; Office of Research and Development, U.S. Environmental Protection Agency, Athens, Georgia, USA;; Office of Research and Development, U.S. Environmental Protection Agency, Athens, Georgia, USA;; Office of Research and Development, U.S. Environmental Protection Agency, Athens, Georgia, USA;

**Keywords:** gridded precipitation, SWAT, watershed modeling, streamflow, calibration

## Abstract

Gridded precipitation datasets are becoming a convenient substitute for gauge measurements in hydrological modeling; however, these data have not been fully evaluated across a range of conditions. We compared four gridded datasets (Daily Surface Weather and Climatological Summaries [DAYMET], North American Land Data Assimilation System [NLDAS], Global Land Data Assimilation System [GLDAS], and Parameter-elevation Regressions on Independent Slopes Model [PRISM]) as precipitation data sources and evaluated how they affected hydrologic model performance when compared with a gauged dataset, Global Historical Climatology Network-Daily (GHCN-D). Analyses were performed for the Delaware Watershed at Perry Lake in eastern Kansas. Precipitation indices for DAYMET and PRISM precipitation closely matched GHCN-D, whereas NLDAS and GLDAS showed weaker correlations. We also used these precipitation data as input to the Soil and Water Assessment Tool (SWAT) model that confirmed similar trends in streamflow simulation. For stations with complete data, GHCN-D based SWAT-simulated streamflow variability better than gridded precipitation data. During low flow periods we found PRISM performed better, whereas both DAYMET and NLDAS performed better in high flow years. Our results demonstrate that combining gridded precipitation sources with gauge-based measurements can improve hydrologic model performance, especially for extreme events.

## INTRODUCTION

Precipitation is a major input for hydrological modeling and streamflow simulation (Tuo et al. 2016). The simulation of watershed processes requires accurate precipitation input that captures the spatial and temporal changes in watershed processes; so that improving the accuracy of precipitation provides better representation of soil moisture, soil water movement, surface runoff, baseflow, and streamflow for accurate simulation of watershed processes (Douglas-Mankin et al. 2010). Precipitation measurements from weather stations may not fully capture spatial and temporal patterns and variability due to low station density. To overcome limitations of gauged data, multiple precipitation sources (gauge, radar, and/or satellite) have been incorporated into gridded datasets (Abatzoglou 2013). These include gauge-only estimates, model-based estimates, ground-based radar estimates, satellite-only estimates, and merged products that represent observed data for input into various hydrologic models and applications. It is important to understand how using these gridded data sources would compare with using gauge data.

One of the most widely used watershed-scale models is the Soil and Water Assessment Tool (SWAT) model (Arnold et al. 1998). SWAT is a semidistributed, process-based, continuous, daily time step watershed-scale model which has been used extensively throughout the world (Gassman et al. 2007; Douglas-Mankin et al. 2010; Tuppad et al. 2011; Arnold, Moriasi, et al. 2012). SWAT was developed in the early 1990s by the United States (U.S.) Department of Agriculture (USDA)-Agricultural Research Service (Arnold et al. 1998; Neitsch et al. 2011; Arnold, Kiniry, et al. 2012) and has undergone continuous review and expansion of its capabilities (Neitsch et al. 2011). SWAT uses weather data, soil properties, topography, land use/cover, and land management to predict the impact of management practices on water, nutrient, sediment, and agricultural chemical yields. SWAT is a robust watershed model (Arnold and Allen 1996; Arnold et al. 1999; Abbaspour et al. 2007; Gassman et al. 2007) and has been used to assess land use/cover effects and climate change influences on water resources worldwide (Carvalho-Santos et al. 2016; Gabriel et al. 2016; Mwangi et al. 2016). In the U.S., SWAT is often used by federal and state agencies to support water resources management (Gassman et al. 2007; Arabi et al. 2008; Douglas-Mankin et al. 2010) and Total Maximum Daily Load development (Borah et al. 2006; Kang et al. 2006). SWAT has been used at various scales from field scale, small watersheds to bigger, regional watersheds (Gassman et al. 2007; Luo et al. 2008; Douglas-Mankin et al. 2010; Tuppad et al. 2011) and different environmental conditions (Gassman et al. 2007). Many different precipitation sources have been used for these applications.

Recent SWAT studies show an increasing trend toward using alternatives to rain gauge networks. Examples include high-resolution radar precipitation such as the National Weather Service (NWS), Next Generation Weather Radar — NEXRAD (Tuppad et al. 2010; Zhang and Srinivasan 2010; Gali et al. 2012; Price et al. 2014; Gao et al. 2017); interpolated gridded datasets from the Parameter-elevation Regressions on Independent Slopes Model (PRISM) from the PRISM Climate Group of Oregon State University (Gao et al. 2017; Radcliffe and Mukundan 2017); and Daily Surface Weather and Climatological Summaries (DAYMET) (Baskaran et al. 2010; Mehan et al. 2017). Other precipitation data sources, which are rarely used in SWAT but have high potential, include the North American Land Data Assimilation System (NLDAS) and the Global Land Data Assimilation System (GLDAS). Nigro et al. (2010) indicated that incorporating NLDAS precipitation as input improved the water quality model performance mainly because NLDAS captures precipitation events accurately.

The type of precipitation data source affects the calibration and the simulation outputs, especially for larger watersheds with complex, heterogeneous terrains. Studies (Moon et al. 2004; Kalin and Hantush 2006; Sexton et al. 2010; Tuppad et al. 2010; Gali et al. 2012; Tobin and Bennett 2013; Gao et al. 2017; Radcliffe and Mukundan 2017) have evaluated the SWAT model parametrization to precipitation data sources, along with how data spatial and temporal resolutions impact simulated streamflow, model calibration, and associated uncertainties. Prior studies (Moon et al. 2004; Kalin and Hantush 2006; Sexton et al. 2010; Tuppad et al. 2010; Gali et al. 2012; Tobin and Bennett 2013; Price et al. 2014; Gao et al. 2017) concluded that there are spatial-scale dependencies for accuracy of model simulation, however, the studies compared only one or two gridded sources to gauged data. Many of these studies calibrated SWAT with monitored precipitation (from National Climatic Data Center [NCDC]) and then ran simulations using the parameters of that SWAT model with gridded precipitation with no further calibration.

Compared to previous work, our study uses four gridded, publicly available, and differently scaled precipitation data sources (DAYMET, GLDAS, NLDAS, and PRISM) and the Global Historical Climatology Network-Daily (GHCN-D) over 25 years (1988–2013). We calibrated each SWAT model using each of the different precipitation sources. This allowed us to account for the impact of each type of data source on the calibration and associated parameter sensitivity. In addition, we began our analysis by comparing gridded and gauged precipitation, using standard statistical measures and extreme precipitation indices (Zhang et al. 2011).

The specific objectives of this study were to: (1) assess how well the datasets (DAYMET, GLDAS, NLDAS, and PRISM) captured precipitation conditions when compared with GHCN-D, based on standard statistical measures and precipitation indices (annual number and maximum consecutive wet and dry days) within the study watershed; (2) evaluate the sensitivity of the SWAT model flow parameters under different precipitation settings; (3) assess the impact of precipitation input on SWAT calibration and validation; and (4) evaluate impacts of gridded precipitation sources on simulations of streamflow and other water balance components.

## METHODS

### Watershed Background

**FIGURE 1.**
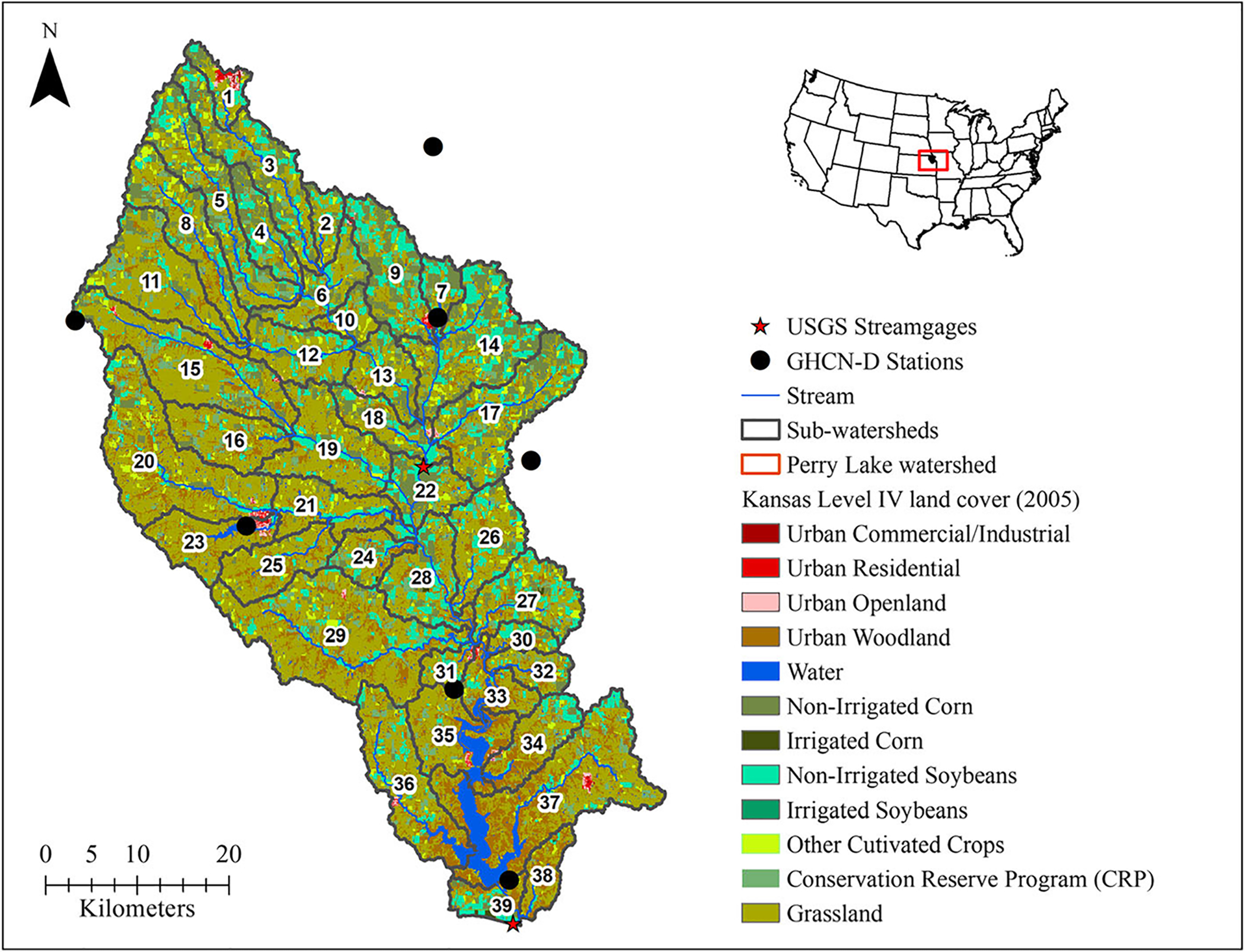
Map of the Delaware watershed at Perry Lake with overlay of National Climatic Data Center (NCDC)- Global Historical Climatology Network-Daily (GHCN-D) stations (precipitation), United States (U.S.) Geological Survey gauge locations used for flow calibration, Soil and Water Assessment Tool (SWAT)-generated reach network, subwatersheds, and dominant land-use based on 2005 Kansas Level IV land cover.

The Delaware watershed at Perry Lake (hydrologic unit code [HUC] 10270103, Figure 1), a HUC-8 level subwatershed of the Kansas River Basin in northeast Kansas, was the study area. It was chosen because of researchers’ familiarity with this agricultural watershed from previous work (Sinnathamby 2014). The site has a drainage area of approximately 2,988 km^2^ that includes one federal reservoir, Perry Lake, managed by U.S. Army Corps of Engineers. The watershed covers parts of five Kansas counties (Atchison, Brown, Jackson, Jefferson, and Nemaha) and extends over parts of two U.S. Environmental Protection Agency (USEPA) level IV ecoregions: Western Corn Belt (83.7%) and Central Irregular Plains (16.3%). The region has an average annual precipitation of 762 mm, and about 82% of that (~625 mm) falls from April to September. Mean annual temperature ranges from 11.1°C to 12.2°C (52°F–54°F) (Sophocleous 1998). Elevation of the watershed ranges from 252 to 428 m, with an average slope of 5.2%. The watershed has mostly (≈77%) fine-textured (silt and clay) soils, dominated by moderately high (hydrologic soil group C) and high (hydrologic soil group D) runoff potentials; it consists primarily of Pawnee clay (30.5%), Grundy silt clay (30.0%), and Kennebec silt (16.1%) soil groups. Agriculture is the dominant land use (68.9%), followed by rangeland (15.4%) and forest land (12.48%); primary crops are hay (32.3%), dryland corn (14.0%), and soybean (13.4%).

### Precipitation Inputs and Processing

Five precipitation data sources (four interpolated and gridded, and one set of measured data) were used in this study. Gridded data included DAYMET, GLDAS, NLDAS, and PRISM. DAYMET (Thornton et al. 1997; NASA-ORNL-DAAC 2018) is a collection of gridded estimates of daily weather parameters, generated by interpolation and extrapolation from daily meteorological observations at a 1 × 1 km spatial resolution for North America. Its interpolation method accounts for topo-climatic factors such as elevation, aspect, slope, distance to coast, and land surface temperature (Oyler et al. 2015).

PRISM (PRISM-Climate-Group 2018) is a regression-based model that uses point meteorological observations, elevation, and other spatial datasets to generate gridded climatic elements (Daly et al. 2002). It was developed by the PRISM Climate Group at Oregon State University, and it provides long-term interpolated climate products from plot- to watershed-scale. PRISM incorporated data from point measurements from multiple networks, including the NWS’s Cooperative Observer Network (COOP). PRISM precipitation and temperature datasets (1981–present) are available throughout the contiguous U.S. at 2.5 arcmin (~4 km) spatial resolution (Daly et al. 2008). Detailed descriptions of PRISM’s algorithms, structure, input grids, and operation can be found in Daly et al. (2002) and Daly et al. (2008).

NLDAS (Xia et al. 2012; NASA-LDAS 2018b) provides higher temporal resolution (hourly) total precipitation in kg/m^2^ at 1/8th-degree grid spacing. It is derived by combining National Oceanic and Atmospheric Administration’s (NOAA) daily National Center for Environmental Prediction Climate Prediction Center gauge-based precipitation analyses and hourly National Weather Service Doppler radar-based (WSR-88D) precipitation analyses (Nigro et al. 2010). GLDAS (NASA-LDAS 2018a) precipitation, however, is globally available at three-hour temporal and 1/4th-degree spatial resolution. It is derived from an uncoupled land surface modeling system that drives multiple models and integrates a large quantity of observation-based data. Detailed information on GLDAS can be found in Rodell et al. (2004).

The most comprehensive source of ground-based observed weather data in the U.S. is the GHCN (Menne et al. 2012). GHCN-Daily, hereafter GHCN-D (NOAA-NCEI 2018), is an integrated database of daily climate summaries from land surface stations across the globe. GHCN-D in the U.S. is a composite of climate records dating back to the 1800s from more than 20 sources, with more than 40,000 stations in the contiguous U.S., which were merged and then subjected to a specialized suite of quality assurance procedures and reviews (Peterson et al. 1998). GHCN-D includes most data from stations operated by COOP stations. As in a recent study (Behnke et al. 2016) we used GHCN-D precipitation data as “reference” data.

**FIGURE 2.**
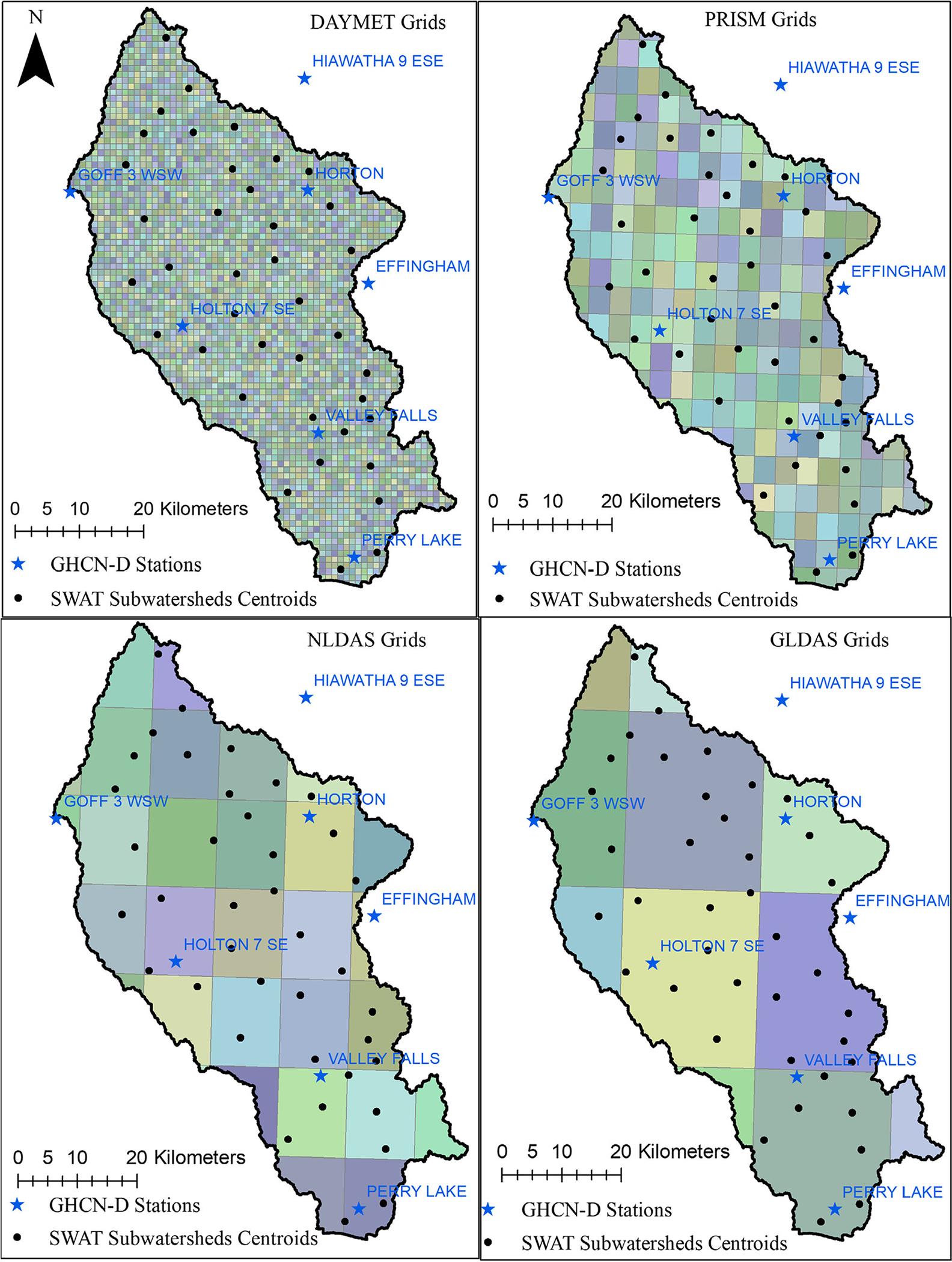
Spatial resolution of precipitation data sources by spatial grids with centroid points of subwatersheds (Note: boundaries and labels of subwatersheds are shown in Figure 1). Figure’s grid illustration is based on (Golden et al. 2010).

A workflow using the Hydrologic Micro Services infrastructure (HMS), an USEPA developed collection of web services, was used to download and process weather data at daily scale (USEPA 2018). Detailed descriptions on HMS workflow can be found in Supporting Information (S1) and brief descriptions of precipitation data sources used in this study are given in Table 1. Spatial coverage and grid resolution of precipitation data sources with centroids of SWAT model subwatersheds are presented in Figure 2.

### Precipitation Comparison

Precipitation comparison was performed to evaluate how well the gridded datasets correlated with GHCN-D data. The analysis compared the GHCN-D and gridded precipitation at GHCN-D location points to better understand parameter sensitivity and the runoff response of the calibrated SWAT model. Daily precipitation from each gridded dataset was extracted only for grid cells that contained GHCN-D stations. As a result, seven GHCN-D stations within or near the study watershed with daily records from 2001 to 2013, were used for the analysis (Figure 2). The analysis years were determined to include similar years of all dataset since GLDAS availability (with the same spatial resolution) starts in 2000 (Table 1). Except for one station (Hiawatha 9 ESE which had 9.3% missing days), there were fewer than 7% missing days in the whole study period (Table 2). Missing days were removed from gridded data during precipitation analysis. Daily precipitation values flagged by quality control procedures for GHCN-D (Durre et al. 2010) were also excluded.

Statistical measures such as standard deviation, correlation coefficient (*r*) and root mean square error (RMSE) were used to assess how well precipitation extracted from each gridded source at GHCN-D gauge locations matched GHCN-D precipitation. A Taylor diagram (Taylor 2001), commonly used in climatology, were used to visually depict the standard measures results. In addition, six precipitation indices checked how well the gridded data captured relatively extreme weather conditions (Table 3); these have been used in many studies (Alexander et al. 2006; Donat et al. 2013; Sillmann et al. 2013; Behnke et al. 2016). Mean bias-based matrix plots were used to compare the performance of gridded products to GHCN-D.

### SWAT Model Development

ArcSWAT 2012, built for ArcGIS 10.3 (ESRI, Redlands, CA), was used to construct a model of the Delaware watershed at Perry Lake with 39 subwatersheds (Figure 1) and 4,161 hydrologic response units with unique land-use, slope, and soil attributes. Soil data were extracted from the State Soil Geographic Dataset soil dataset in the SWAT database; slope was derived from a 30-m digital elevation model (DEM); and land cover was calculated from the 2005 Kansas Level IV land cover map developed by Kansas Applied Remote Sensing Program. Cropland Data Layer from the USDA National Agricultural Statistics Services from 2008 to 2010, and the National Land Cover Database layer from 2006 were overlaid to determine dominant crop rotations (Srinivasan et al. 2010). Detailed model setup can be found in Sinnathamby (2014).

Following initial SWAT model setup using the same model inputs, five SWAT models (GHCN-D SWAT, DAYMET SWAT, GLDAS SWAT, NLDAS SWAT, and PRISM SWAT) were created by incorporating the respective precipitation sources. We did this because we are interested in how each SWAT model performs using solely the specified precipitation dataset. In the GHCN-D SWAT model, seven GHCN-D stations located within or near the Delaware watershed (Figures 1 and 2), with data for the reference period (1983–2013), were used. Four other sources of gridded precipitation were downloaded using USEPA’s HMS workflow by providing centroids of the SWAT subwatersheds; geographic locations of the centroids are shown in Figure 2. These were used to download precipitation data because SWAT takes one weather station input per subwatershed — thus, 39 points representing centroids of each SWAT subwatershed were used. In the case of NLDAS and GLDAS, multiple subwatersheds’ centroids were on the same grid cell of the precipitation source, whereas centroids in DAYMET and PRISM were on different spatial grids (Figure 2). This enabled the SWAT models for DAYMET and PRISM to capture more spatial variability when compared with the SWAT models for NLDAS and GLDAS. Four other weather variables at daily scale (temperature, solar radiation, relative humidity, and wind speed) were generated by SWAT.

### SWAT Sensitivity Analysis, Calibration, and Validation

Calibration and validation are routine steps in watershed modeling that assess performance and confirm a model’s readiness for further analysis. Sensitivity analysis helps to characterize variation of model input factors on model output and identifies influential parameters. It also guides model calibration and validation and informs how to prioritize efforts to reduce uncertainties (Norton 2015; Pianosi et al. 2016). The first five years of the total simulation period (January 1983–December 2013) were a warm-up to allow the model to reach hydrologic equilibrium and were excluded from the analysis. The 13-year calibration period was from January 1, 2001 to December 31, 2013; selected since all precipitation sources were available. The 13-year validation period ran from January 1, 1988 to December 31, 2000. Calibration was carried out using a monthly time-step at the outlet of a headwater stream (Delaware near Muscotah, U.S. Geological Survey [USGS] 06890100); the watershed outlet (Delaware at Perry Lake, USGS 06890898); and the reservoir outlet using SWAT Calibration and Uncertainty Program (SWAT-CUP). Daily flow at the reservoir outlet was obtained from the U.S. Army Corps of Engineers Kansas City office.

SWAT-CUP has different tools for calibration, sensitivity analysis, and uncertainty analysis. These include: Sequential Uncertainty Fitting ver. 2 (SUFI-2); Particle Swarm Optimization; Generalized Likelihood Uncertainty Estimation; Parameter Solution; and Markov Chain Monte Carlo algorithms (Abbaspour 2015). SUFI-2, a widely used calibration and uncertainty analysis procedure, was used for sensitivity analysis and calibration. It has been recommended as an efficient program for large-scale models (Yang et al. 2008; Abbaspour et al. 2015) and has also been identified as achieving good prediction of uncertainty ranges using a reasonable number of data points with the fewest runs (Yang et al. 2008). Global sensitivity analysis (GSA) available under SUFI-2 helps to rank input parameters by relative influence on the model output, based on the *t*-stat and *p*-value statistics. The *t*-stat is the coefficient of the parameter divided by its standard error (Abbaspour 2015): the larger the *t*-stat (absolute value), the more sensitive the parameter. The *p*-value measures the significance of the sensitivity of that parameter. Ranking parameters by significance enhances model understanding and identifies the most important controls of model behavior (van Werkhoven et al. 2008; Matott et al. 2009).

For each of the five models, the automated calibration process was conducted with an identical range of parameter values and calibration/validation periods for comparison purposes. Detailed description of the 21 parameters used in calibration is shown in Table 4. Automated calibration ensures consistency of the process for all models and minimizes the modeler bias in calibration exercises conducted for different precipitation sources. Similar procedures were followed in other recent studies (Bitew et al. 2012; Tobin and Bennett 2013; Yang et al. 2014; Radcliffe and Mukundan 2017; Ren et al. 2018). Initial parameter ranges were selected based on professional judgment and literature. The precipitation source assessment was evaluated by assessing the ability of the model to reproduce observed streamflow. Through individual sensitivity analysis and calibration, each precipitation source was given an equal chance to adjust relevant sensitive parameters and converges different parameter intervals to match observed flow.

Each model executed 500 simulations for each autocalibration iteration. An initial 300–500 simulations are recommended for studying model performance and for regionalizing parameters (Arnold, Moriasi, et al. 2012). At the end of an iteration with 500 simulations, parameter sensitivities were determined through GSA. Only one iteration was used to avoid re-calibration using a different range of parameter values for each model in the subsequent calibration. The Nash- Sutcliffe efficiency (NSE) was used to estimate model performance during calibration (Nash and Sutcliffe 1970) since it is a commonly used statistical measure in SWAT studies (ASCE 1993; Moriasi et al. 2007).

In addition to the sensitivity analysis, a visual inspection of the simulated inputs and the relative changes in NSE were analyzed to identify parameter distributions over precipitation sources and model performance using loess plots (Cleveland et al. 1991). One-way ANOVA and Tukey multiple pairwise-comparisons were used to identify performance differences between each precipitation dataset. A similar procedure was carried out with the 20 best calibration sets of parameters to verify precipitation source performance during validation. This helped to control uncertainty and ensure the autocalibration was not randomly fitting parameters, and that simulated streamflow was not statistically significant.

NSE, coefficient of determination (*R*^2^), and Kling–Gupta efficiency (KGE) (Gupta et al. 2009) were used as model evaluation statistics. These are standard regression statistics in watershed modeling (Moriasi et al. 2007). NSE is a normalized index that measures the magnitude of residual variance, compared to observed variance (Nash and Sutcliffe 1970; Moriasi et al. 2007). NSE ranges between −∞ and 1, with 1 being ideal. *R*^2^ describes the degree of linear relationship between observed and model output. *R*^2^ ranges from 0 to 1. NSE and *R*^2^ are sensitive to high streamflow values during storms (Krause et al. 2005; Moriasi et al. 2007; Moriasi et al. 2015). To overcome this issue, we included KGE. KGE is the goodness-of-fit measure developed by Gupta et al. (2009), which provides decomposition of NSE and mean squared error; KGE facilitates the analysis of relative importance of correlation, bias, and variability in hydrologic modeling (KGE-hydroGOF 2017). KGE ranges from −∞ and 1. The closer the value to 1, the more accurate the model is. The RMSE-observations standard deviation ratio (RSR), an error index statistic, was also used in model evaluation. For stream flow, Moriasi et al. (2015) proposed NSE values > 0.50 and *R*^2^ > 0.60 to be a satisfactory level for monthly scales. A KGE value > 0.50 (Gupta et al. 2009) and RSR value < 0.60 are considered satisfactory (Moriasi et al. 2007). In addition, NSE values > 0.65, *R*^2^ > 0.80, KGE values > 0.60, and RSR value < 0.50 are considered satisfactory at an annual scale in this study.

Two other SWAT-CUP performance measures (*P*-factor and *R*-factor) were used to indicate the strength of model calibration and uncertainty assessment (Arnold, Moriasi, et al. 2012; Abbaspour et al. 2015). Abbaspour et al. (2015) defined the *P*-factor as the percentage of measured data covered by 95% prediction uncertainties (95PPU). It measures the ability to capture uncertainties, and its value ranges from 0 to 1, where 1 indicates that 100% of the observed data are covered by 95PPU. The *R*-factor indicates thickness of the 95PPU, since it is the ratio of the average width of the 95PPU band and the standard deviation of observed data; a lower value of the *R*-factor is better. A *P*-factor value > 0.7 and *R*-factor < 1.5 are recommended for flow modeling (Abbaspour et al. 2015) and used to measure prediction uncertainty.

## RESULTS AND DISCUSSION

### Comparison of GHCN-D and Gridded Precipitation Data

**FIGURE 3.**
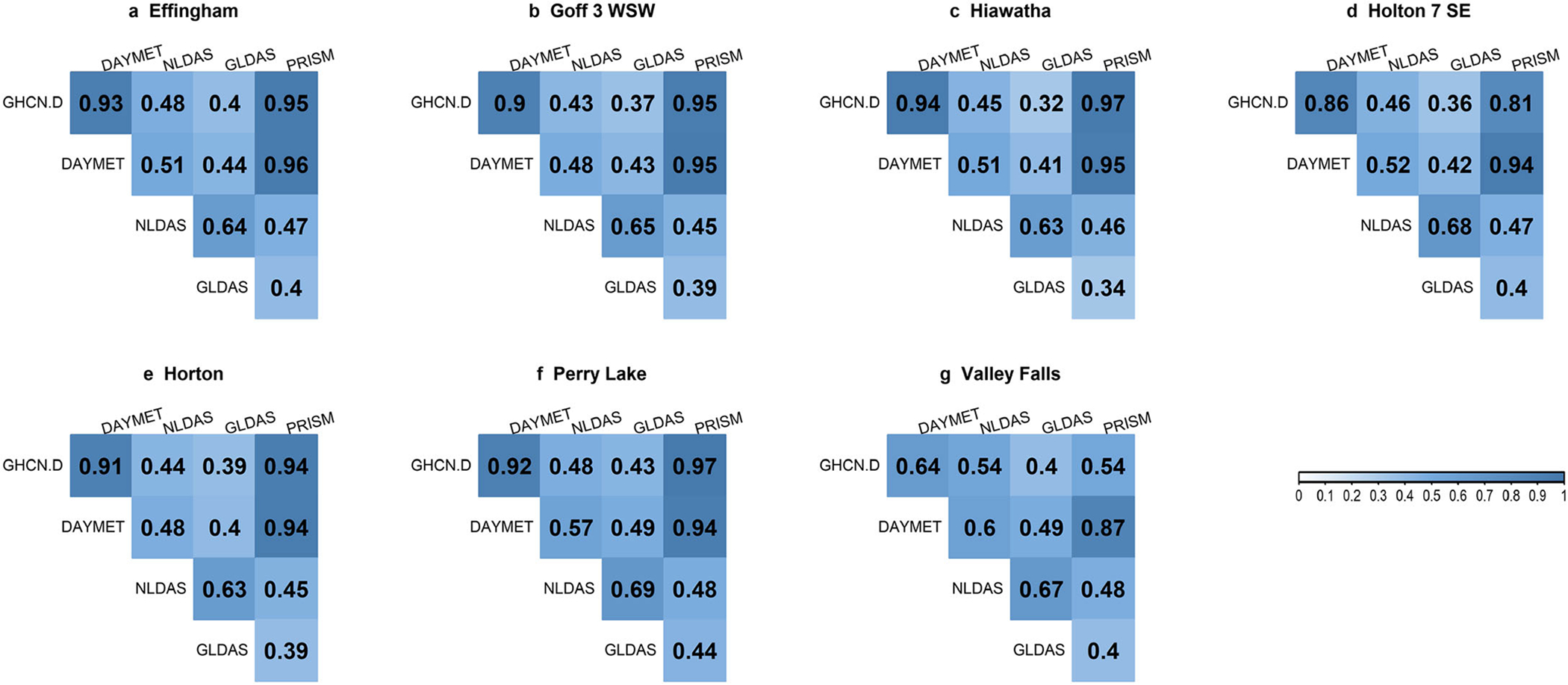
Correlogram of the precipitation correlation matrix for each station (a–g). Positive correlations at significance level = 0.05 are displayed in blue. Color intensity and size are proportional to the correlation coefficients.

The four gridded precipitation data sources showed different relationships when compared with reference data (GHCN-D). The correlation matrix of the four sources vs. GHCN-D at seven locations is shown in Figure 3: darker blue shows the better correlations. For all stations except Valley Falls, DAYMET and PRISM precipitation matched GHCN-D weather station records well, with high correlation coefficients (>0.81, most > 0.94) (Figure 3). Five of the seven stations indicated the highest correlation coefficients for GHCN-D and PRISM from 2001 to 2013. DAYMET was the second-best fit for GHCN-D. Valley Falls had the weakest correlation of gridded sources; DAYMET and GHCN-D had a 0.64 correlation coefficient and PRISM and GHCN-D had 0.54, whereas NLDAS and GLDAS show much weaker correlations (<0.50). With lower spatial resolution (Figure 2), GLDAS had the largest discrepancies and lowest correlation coefficients. Both Valley Falls and Perry Lake lay in the same grid of GLDAS, and neither matched GHCN-D observed data well.

**FIGURE 4.**
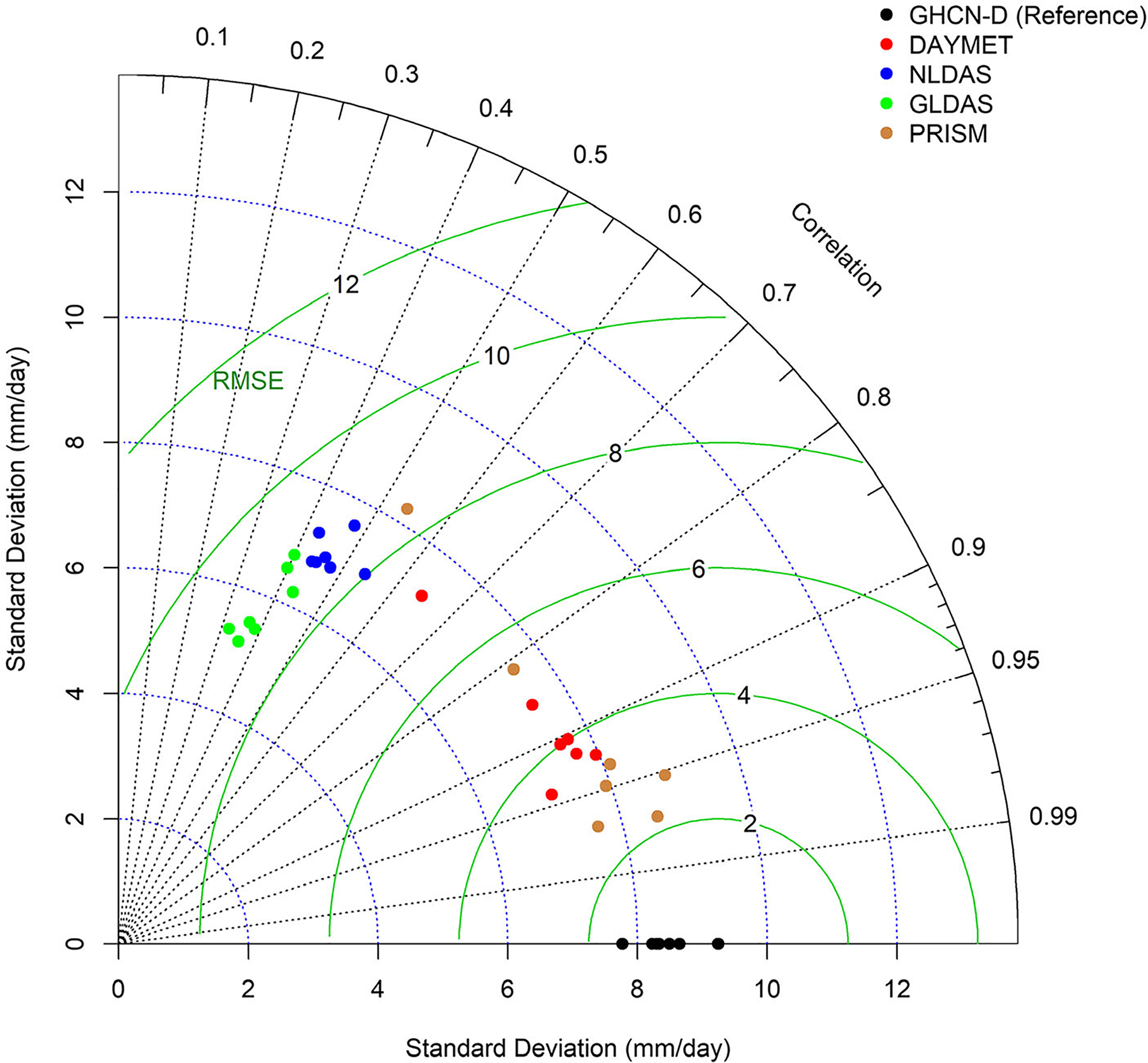
Taylor diagram showing the ability of precipitation datasets to represent GHCN-D based on daily precipitation (2001–2013).

Correlation results were supported by multiple statistical measures depicted by the Taylor diagram (Figure 4). Based on the standard statistical measurements presented in the figure, data for five PRISM precipitation stations provided the best match with GHCN-D, in terms of correlation coefficient, standard deviation (daily variability), and RMSE (Figure 4). Those PRISM stations indicated very similar variability (standard deviation) to the GHCN-D stations. After PRISM, DAYMET precipitation was also displayed very close values. Two other stations of PRISM precipitation, Valley Falls and Holton 7 SE, exhibited similarity to DAYMET, however, DAYMET was less similar to GHCN-D than other gauge stations. Data products that exhibited lower correlation coefficients (<0.70) showed much larger RMSE (Figure 4). A similar observation was found by Behnke et al. (2016), who also reported a larger mean absolute error for NLDAS than DAYMET and PRISM for the Prairie ecoregion where the study area is located.

Since GHCN-D, DAYMET, and PRISM were originated using COOP stations, a close resemblance was expected between them (Thornton et al. 1997; Daly et al. 2002). Golden et al. (2010) also made similar observations between NCDC COOP stations and PRISM precipitation from 2001 to 2003. The influence of spatial resolution may also play a part in these discrepancies. Results presented above show that both precipitation datasets with relatively higher resolution, DAYMET and PRISM (Figure 2), agreed best overall with GHCN-D observations, whereas NLDAS and GLDAS had the largest discrepancies. This is noteworthy because both of NLDAS and GLDAS datasets have relatively coarser spatial resolution (Figure 2).

**FIGURE 5.**
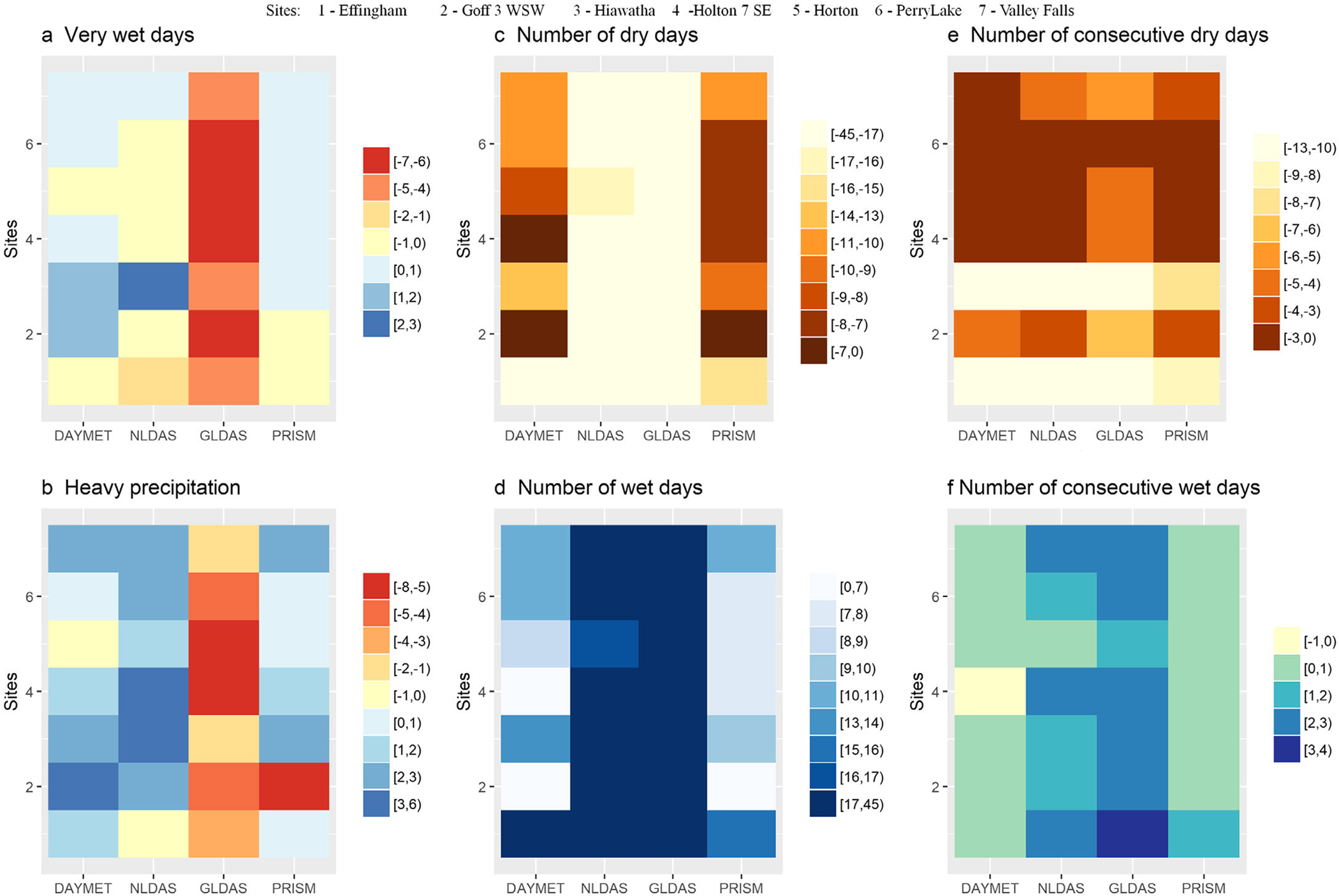
Mean bias of precipitation indices (a–f) for stations and precipitation source. These values are calculated compared to observed values (GHCN-D). Note: Positive values show overprediction and negative values show underprediction compared to GHCN-D.

A similar situation was observed in analyzing precipitation indices: matrix plots of mean bias of precipitation indices focused on relatively extreme weather conditions (compared to GHCN-D) are shown in Figure 5. Results indicate that there were fewer number dry days and fewer number of consecutive of dry days for all gridded precipitation data sources compared to GHCN-D (Figure 5c and 5e). The number of dry days were fewer by 15 or more — up to 45 — days in NLDAS and GLDAS data, across the sites. The number of consecutive dry days were also fewer across data sources. Across stations, PRISM showed closer values of dry day-related indices to GHCN-D, followed by DAYMET. More number of wet days and number of consecutive wet days were observed in GLDAS (Figure 5d and 5f). In the case of very wet days (precipitation ≥ 95th percentile), PRISM and DAYMET had slightly higher; NLDAS had slightly fewer; and GLDAS had fewer days, compared to the GHCN-D (Figure 5a). For heavy precipitation (precipitation ≥ 10 mm), however, PRISM (except for one site), DAYMET, and NLDAS had slightly higher; and GLDAS had fewer days. Consistent with the Taylor diagram, PRISM (mean bias ≤ ±2.3 days) and DAYMET (mean bias ≤ ±3.6 days) most closely matched GHCN-D observations in the number of very wet days, days with heavy precipitation, and number of consecutive wet days. Differences between GHCN-D and gridded data sources in dry and wet number of days may indicate that gridded data sources are capturing localized rainfall events that GHCN-D missed. Price et al. (2014) and Radcliffe and Mukundan (2017) reported that gauges underestimated rainfall in large storms compared to radar data. Negative bias of rain gauges during heavy precipitation may be due to water loss caused by wind and erratic behavior of mechanical aspects of the gauge (Molini et al. 2005; Lanza and Stagi 2008).

### Parameter Sensitivity under Different Precipitation Settings

**FIGURE 6.**
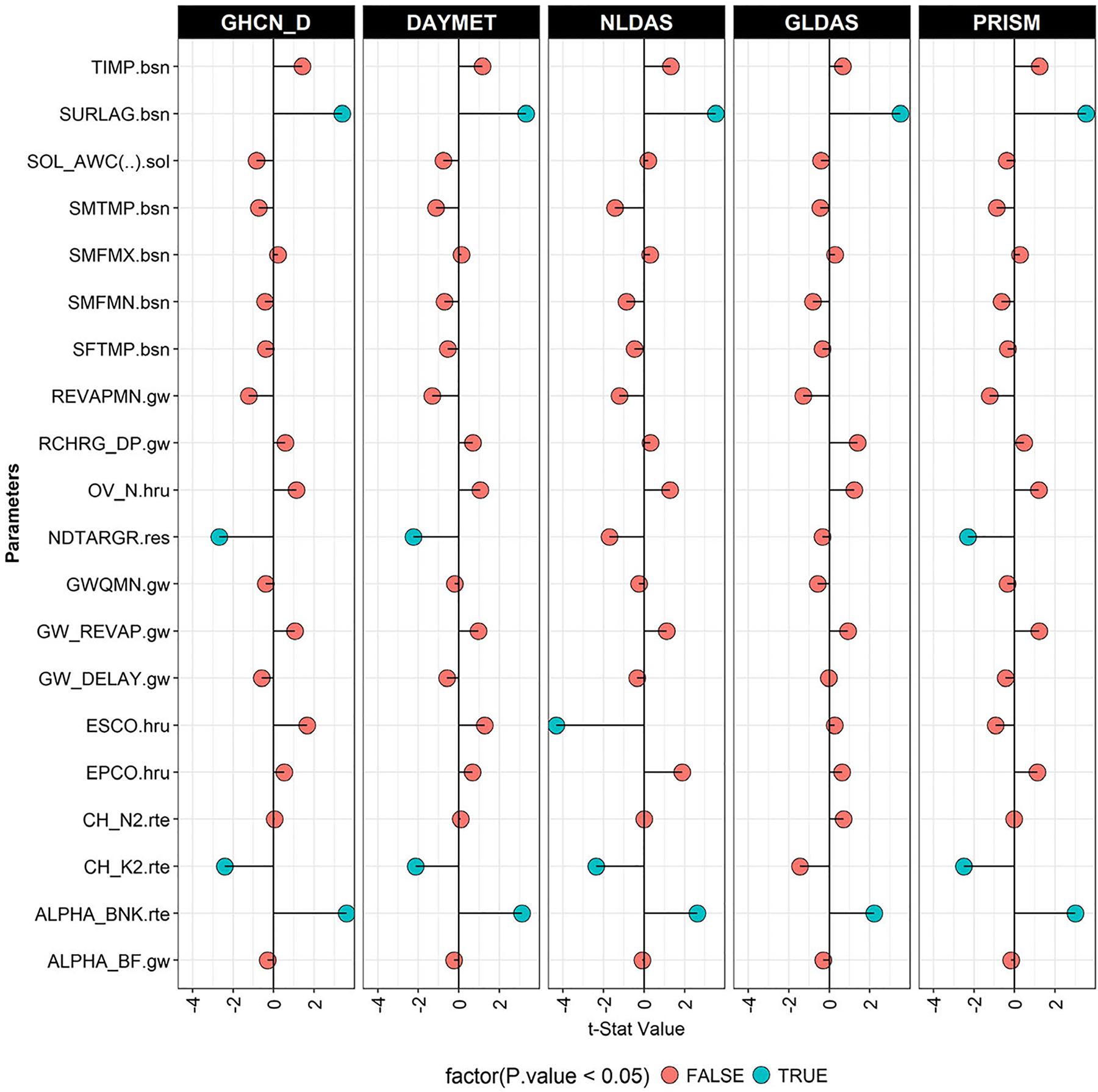
Sensitivity of parameters during calibration period. The highest sensitive parameter, CN2 was removed from the plot to identify the outlying observation. The CN *t*-stat ranges from −11.1 to −19.6. The lowest negative value was observed with GLDAS and the highest was in NLDAS. DAYMET, GHCN-D, and PRISM had −12.8, −13.9, and −16.3, respectively. Note: True means statistically significant and false means not significant based on *p*-value of 0.05.

The parameters used in calibration and their sensitivities are shown in Table S1 and Figure 6. The SCS curve number (CN2); baseflow alpha factor, or recession constant, for bank storage (Alpha_Bnk); and surface runoff lag coefficient (SURLAG) were similar and the most sensitive (*p* ≤ 0.03) parameters in all models. Effective saturated hydraulic conductivity in main channel alluvium (CH_K2) was sensitive in GHCN-D SWAT, PRISM SWAT, DAYMET SWAT, and NLDAS SWAT models (*p* ≤ 0.05). Number of days to reach target storage from current reservoir storage (NDTARGR) was also identified as one of the five most sensitive parameters for GHCN-D, DAYMET, and PRISM SWAT models (*p* ≤ 0.05). Soil evaporation compensation factor (ESCO) was sensitive in NLDAS (*p* ≤ 0.05). Even though it is difficult to point out specific reasons why each parameter is sensitive in one model and not the other due to the complexity of hydrologic systems, it is believed that it is related to the differences in each precipitation in capturing different extreme conditions and related uncertainties. Ren et al. (2018) and Tuo et al. (2016) also showed that different precipitation inputs affect parameter selection, the best estimate of a parameter, as well as its uncertainty range.

Sensitivity of certain parameters varied for different precipitation sources; and that could be explained by the differences in several indices described in the precipitation comparison analysis (Section Comparison of GHCN-D and Gridded Precipitation Data). As explained earlier, NLDAS was wetter than DAYMET and PRISM, and NLDAS showed higher deviation by underestimating dry days, and overpredicting heavy precipitation, which resulted in higher sensitivity of the CN2. The lowest CN2 was observed with GLDAS SWAT. All models reduced the CN2 (negative t-stat value) (Table 5), which shows all SWAT models are yielding higher runoff than the observed runoff and adjusting CN2 to account for a different mean rainfall from precipitation sources. Reductions of CN2 in the GHCN-D, PRISM, and DAYMET SWAT models (from 5.88% to 7.72%) were higher than NLDAS and GLDAS SWAT models (1.16% and 3.96%, respectively). This may be due to the higher number of very wet days in GHCN-D, PRISM, and DAYMET compared to NLDAS and GLDAS (Section Comparison of GHCN-D and Gridded Precipitation Data). The larger negative relative change in CN2 would result in the largest runoff reduction in the GHCN-D, PRISM, and DAYMET SWAT models, relative to NLDAS SWAT.

The parameter Alpha_BNK characterizes the bank storage recession curve. The higher Alpha_BNK value observed for the NLDAS SWAT model reveals flatter recessions than do the GHCN-D and DAYMET SWAT models. The lowest value observed in GLDAS SWAT denotes a steep recession. Lower SURLAG, compared to the default value (which is 4.0 and considered to represent the average fraction of surface runoff contribution) in GHCN-D, DAYMET, and NLDAS SWAT models show lowered contribution of surface runoff to the main channel. PRISM and GLDAS show (Table S1) higher SURLAG values than the default which reveal higher model contributions of surface runoff to the main channel when these precipitation data are used (Neitsch et al. 2011). This may allow GLDAS and PRISM SWAT models to function better in low flow periods. Higher CH_K2 in GHCN-D, PRISM, and DAYMET SWAT models show more reduction in discharge through recharging groundwater than the NLDAS model. A higher number of days to reach target storage from current reservoir storage (NDTARGR) values than default values (one day) reveal higher reservoir storage. Even though most sensitive parameters are the same in most cases, their best values and uncertainty ranges of parameters were different with different precipitation inputs (Ren et al. 2018).

The five most sensitive parameters (*p* ≤ 0.05) were the same for GHCN-D, DAYMET, and PRISM SWAT models, showing these precipitation sources have similar influence on parameter sensitivity and selection (Tables 5 and S1). This was expected as these three precipitation sources show higher correlation, lesser standard deviation, and close resemblance in predicting precipitation indices (Figures 3–5). More interestingly, both GHCN-D and DAYMET SWAT models had the same “best” fitted values, suggesting very close resemblance between the two precipitation sources. Again, this similarity between GHCN-D, DAYMET, and PRISM SWAT model outputs could be related to their precipitation original sources and how they were modeled. Another critical reason for the close match between GHCN-D, DAYMET, and PRISM is the spatial resolution. ESCO parameter selection in the NLDAS SWAT model may also better represent evapotranspiration (ET) for this precipitation source.

**FIGURE 7.**
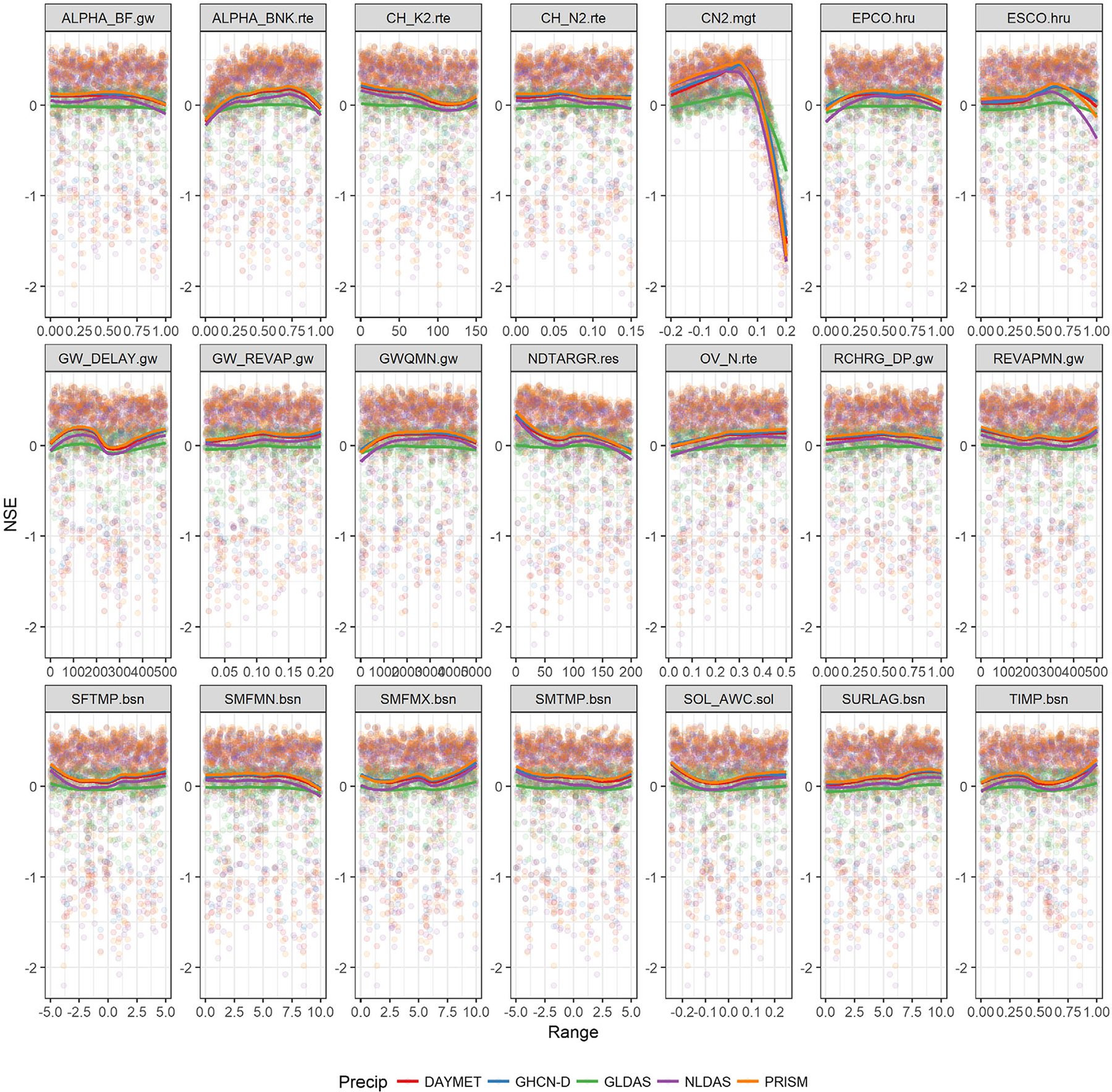
Scatter plots of likelihood values of 500 simulations along with the variation in parameters and their distributions for all precipitation sources during calibration period. Parameter distributions were smoothed using default “loess” method. NSE, Nash–Sutcliffe efficiency.

The relative changes in parameters vs. NSE, during 500 simulations for all precipitation sources, is shown in Figure 7. Distribution of parameters and parameter sensitivity can be identified using these plots. The curve number (CN2) is identified as the most sensitive parameter, with best fitting values of <0.1 in relative change. The figure also shows that parameter distribution is very similar for all precipitation sources, especially DAYMET, GHCN-D, and PRISM. NLDAS also has a close distribution. GLDAS has a different distribution than other sources. Results obtained from Tukey multiple comparisons of means also show that all SWAT models, except GLDAS, are statistically similar for both calibration and validation periods. (Table S2). These results, along with *P*- and *R*-factors observed for calibration and validation, suggest that all precipitation models have acceptable prediction uncertainty and reasonable adjustment of parameters.

These results clearly show that precipitation data sources affect both sensitive parameters and their corresponding ranges of values for the study area with a specific study period. Similar regional studies need to note that parameters sensitivity and ranges of values would not be similar for studies with different data sources and study years. This demands the importance of a cautious approach when studies that utilize literature information to determine calibration parameters for given study areas or for studies in ungauged studies that utilize regional calibrated values.

### Effect of Precipitation Input on SWAT Calibration and Validation

Summary statistics obtained through calibration and validation processes at monthly and annual scales are presented in Tables 6 and S3. All statistical criteria for satisfactory model performance described in Methods were met with GHCN-D, PRISM, DAYMET, and NLDAS SWAT models for calibration and validation periods, and at monthly and annual scales (except *R*^2^ for monthly flow with NLDAS during calibration) at the Delaware River near Muscotah. All statistical criteria for satisfactory model performance were also met with GHCN-D, PRISM, DAYMET, and NLDAS SWAT models at all three calibration sites, except KGE at the reservoir outlet with PRISM SWAT. GLDAS SWAT failed to meet satisfactory conditions at both temporal scales during calibration and validation. Overall, the Delaware River near Muscotah (the upstream watershed) met criteria with higher statistical results with all precipitation sources except GLDAS SWAT. *P*-factor values > 0.70 and *R*-factor values < 1.5 in all conditions indicated adequate strength of model calibration and uncertainty assessment of this study. Using GHCN-D and DAYMET SWAT models resulted in a better fit for both monthly and annual streamflow simulations for calibration and validation periods, with satisfactory values for NSE, *R*^2^, and RSR for all sites (Table S3). NLDAS SWAT also met satisfactory conditions and performed equally well at the annual scale. In the case of the Delaware River at Perry Lake and the reservoir, DAYMET SWAT performed better with higher NSE, RSR, and *R*^2^ at the monthly scale, whereas NLDAS SWAT had a higher KGE. At both sites, however, the *P*-factor was always slightly higher with DAYMET SWAT (Table 6). It is also worth mentioning that even though several model evaluation statistics (NSE, *R*^2^, KGE, RSR, *P*-factor, and *R*-factor) used in subsequent analysis, the initial autocalibration used a commonly applied single statistical measure (NSE) in SWAT studies (ASCE 1993; Moriasi et al. 2007).

**FIGURE 8.**
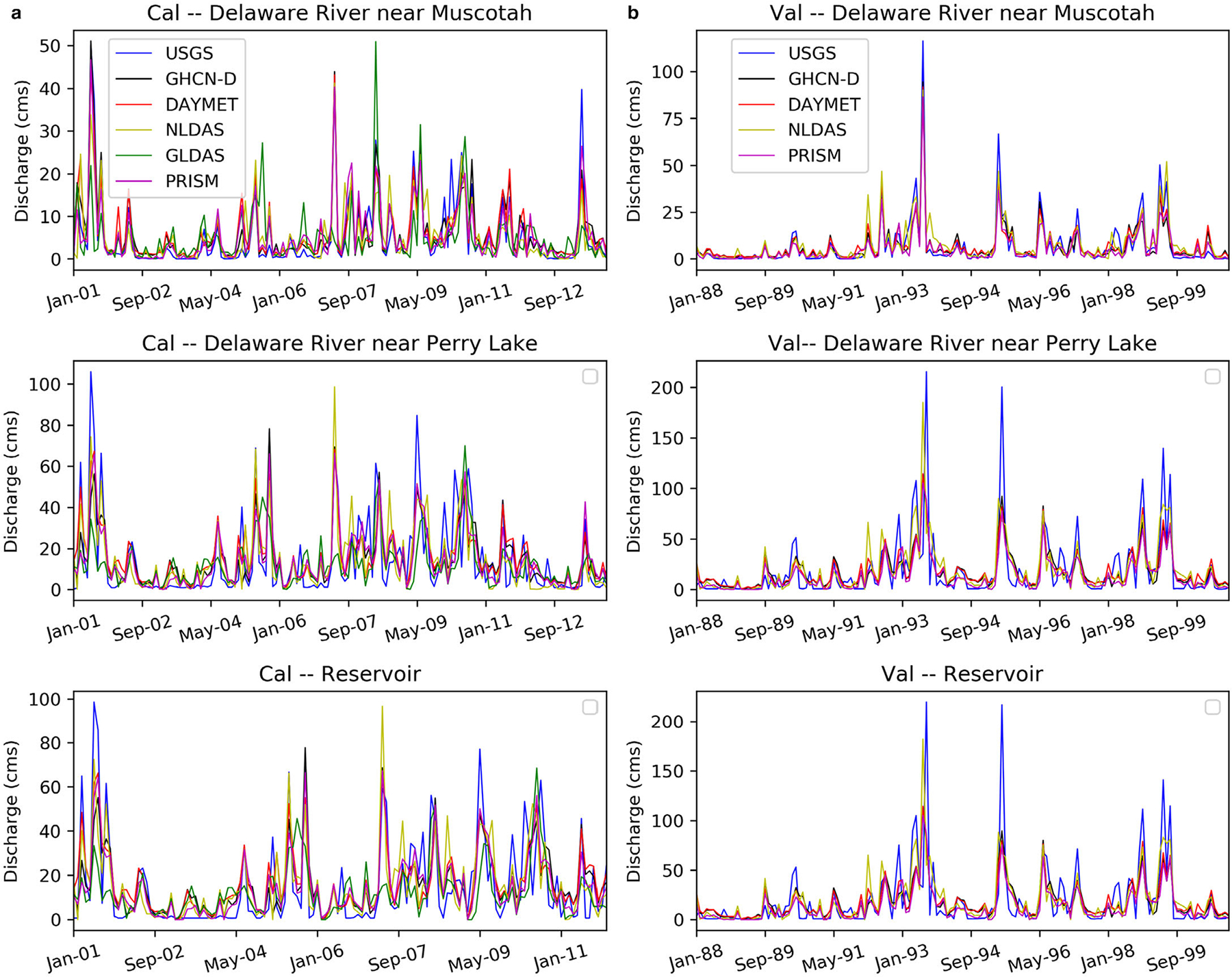
GHCN-D, DAYMET, NLDAS, and GLDAS × SWAT model simulations for the (a) calibration (2001–2013) and (b) validation (1988–2000) period for all three sites.

Results showed that when the number of stations in the watershed increased and there were fewer missing days, GHCN-D SWAT captured the natural variability in the streamflow better than any SWAT model with other gridded precipitation sources used here (Table 6 and Figure 8). In the case of the Delaware River near Muscotah, three stations (Horton, GOFF 3 WSW, and Hiawatha) represented precipitation for that local region. Horton, which had 99.1% precipitation data, covered more than 50% of the subwatersheds. The Delaware River at Perry Lake and the reservoir were covered by Valley Falls and Perry Lake. Valley Falls had only 93.3% data coverage and Perry Lake had 98.2% data coverage. Valley Falls also had continuous missing data for more than 260 days. In that case, DAYMET SWAT outperformed GHCN-D SWAT at the monthly scale and NLDAS SWAT outperformed all other precipitation sources incorporated into models at an annual scale. It is also true that the flow of the Delaware River at Perry Lake is highly influenced by the reservoir operation (Figure 8), since the reservoir outlet is only about 6.5 river kilometers above the calibration point (Figure 1).

DAYMET data are projected on a denser grid (1 × 1 km) than PRISM (~4 × 4 km), NLDAS (1/8th degrees, ~14 × 10.5 km) and GLDAS (1/4th degrees, ~28 × 21 km for the study area). This gave DAYMET an advantage in reflecting the spatiotemporal variability of precipitation, whereas NLDAS and GLDAS were coarser in providing accurate precipitation for a relatively small area (Figure 2). Also, GHCN-D collection at NOAA National Center for Environmental Information has been used as spatially referenced ground observations input to DAYMET, which meant DAYMET closely resembled GHCN-D. The effect of resolution on SWAT simulation can be seen in monthly simulations, however, annual simulations were not influenced by the precipitation source resolution (Tables 6 and S3). GHCN-D, DAYMET, and NLDAS SWAT models performed in a similar manner on an annual scale in this watershed, which shows users can select appropriate precipitation source model based on goal and temporal scale requirements.

### Evaluation of Impacts of Precipitation on Flow and Other Parts of the Water Balance Simulation

**FIGURE 9.**
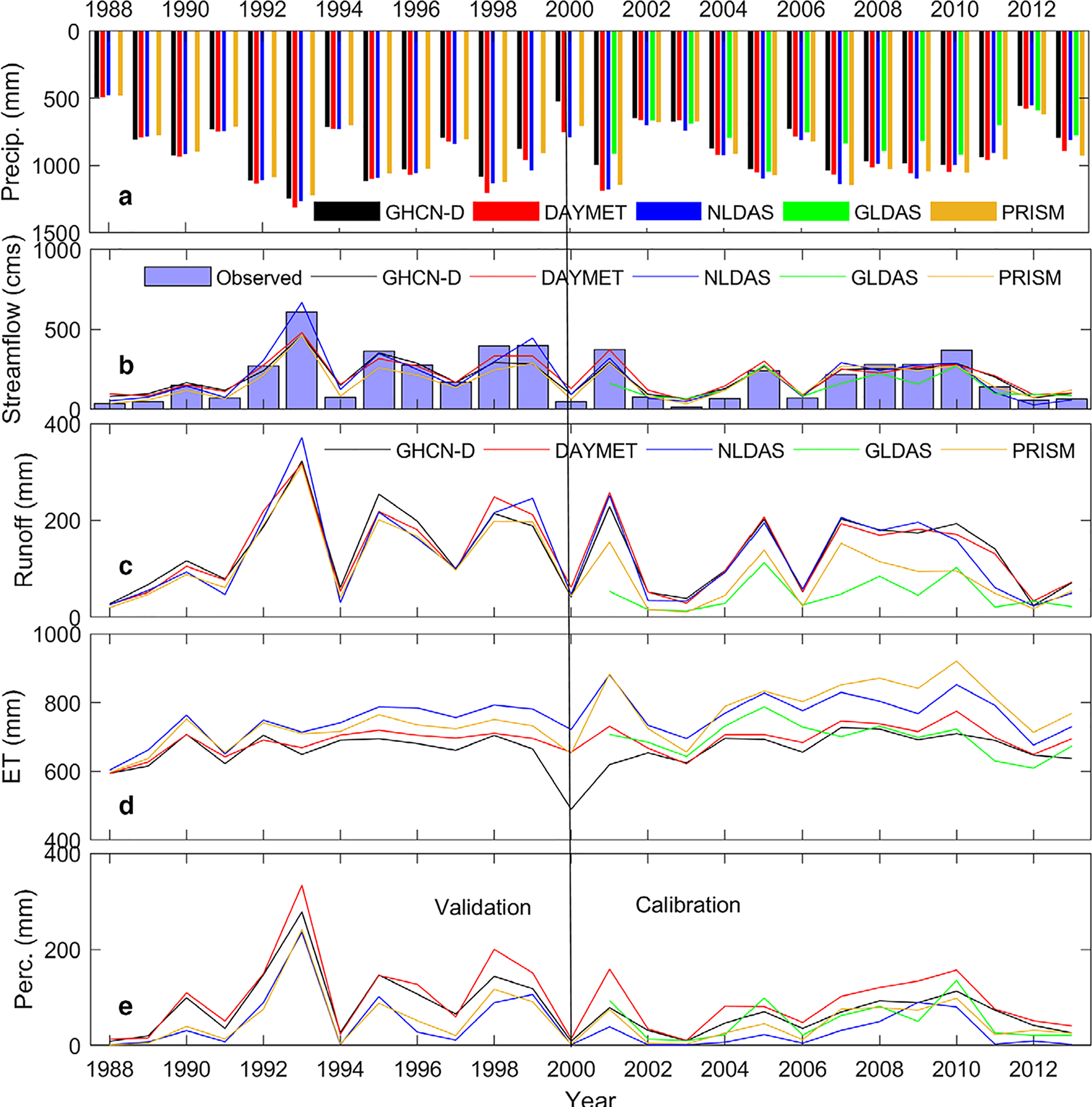
SWAT annual hydrologic balance components with precipitation data sources for Delaware watershed at Perry Lake: (a) annual precipitation, (b) simulated annual streamflow (primary axis) and annual precipitation (secondary axis), (c) surface runoff, (d) evapotranspiration (ET), and (e) percolation to the soil of five precipitation sources are shown.

Major hydrologic components from different precipitation data sources at watershed scale are presented in Figure 9. DAYMET, PRISM, and NLDAS SWAT models overpredicted streamflow compared to GHCN-D (and GLDAS SWAT underpredicted), during dry years when precipitation was less than the annual average flow of the study area. This was expected since DAYMET, PRISM, and NLDAS showed fewer dry days and slightly higher very wet days, heavy precipitation, number of wet days, and number of consecutive dry days over GHCN-D. These conditions resulted in higher ET for these precipitation source models compared to GHCN-D, mainly because real ET components are directly related to water availability in the SWAT model. Higher ET with higher precipitation was reported by Ren et al. (2018). Gao et al. (2017) also found higher ET in gridded source precipitation models than the SWAT GHCN-D model. However, more research is needed to determine the factors why models with gridded precipitation sources deliver higher ET. Although GLDAS exhibited a greater number of wet days and number of consecutive wet days, GLDAS SWAT underpredicted streamflow since it also had fewer very wet days and heavy precipitation days (Figure 5). Surface runoff in most cases paralleled streamflow patterns; underpredicted surface runoff was observed in GLDAS and PRISM SWAT models during validation. Water balance defines a dynamic threshold moisture deficit where additional water becomes excess and contributing to runoff and/or percolating deeper to the soil profile (Easton et al. 2011). The effect of precipitation dataset is also noticed with percolation. The higher percolation was observed with the higher precipitation and therefore percolation follows the precipitation trend. Also, negative correlation was observed between percolation and ET. This was expected as SWAT calculate percolation as a function of soil moisture content (Tripathi et al. 2006).

All 26 simulation years were divided into low or high flow years, based on the Delaware River near Muscotah average annual flows. For low flow years, in which streamflow is < 90% total average annual flow, GHCN-D, DAYMET, and GLDAS SWAT models overpredicted streamflow (Table 7). With slight deviations from observed streamflow, PRISM and NLDAS SWAT models predicted better than models with other sources during low flow years. Even though GHCN-D and DAYMET SWAT models captured temporal variability (Table 7, *R*^2^ values), PRISM SWAT had matched better with observed streamflow during low flow periods. This is mainly because of adjustment of SURLAG and CH_K2 during PRISM SWAT calibration. Radcliffe and Mukundan (2017) also found that the PRISM model performed better during low flow periods compared to NCDC (GHCN-D) model, mainly by including more groundwater recharge parameters during calibration. In wet flow years (flow > 110% average annual flows), all SWAT models underpredicted streamflow (Table 7), with DAYMET and NLDAS most closely resembling the observed streamflow. These specific nature of better simulating dry flow and high flows of PRISM and DAYMET and NLDAS can be effectively used in low flow and high flow specific scenarios such as the effect of low flow on aquatic ecosystem in riverine ecology and nonpoint control planning during high flow events.

These results show the potential in using gridded precipitation for hydrological modeling. With densely populated stations at the regional scale, it is preferable to use monitored data when complete data are available for the study period. Few wide-area gauge monitoring networks with dense, continuous data exist, however, especially at larger spatial scales. The gridded dataset is advantageous because it provides continuous data at spatial and temporal scales across the continental U.S., and for longer periods. Results also showed that gridded precipitation performed well in capturing extreme weather conditions, for example, wet and dry flows — even better than with monitored data. The recent availability of large-scale precipitation grids in a consistent format and improved technology have facilitated the use of multiple gridded data in hydrological studies. These grids can be combined by blending desirable attributes and daily gauge-based precipitation for better model predictions, especially when extreme events are of critical concern.

## CONCLUSION

This study evaluated the ability of four spatially gridded datasets (DAYMET, GLDAS, NLDAS, and PRISM) to represent precipitation compared to GHCN-D as a reference. For the analysis, the SWAT model was configured for a 2,988 km^2^ Delaware watershed at Perry Lake in northeastern Kansas with similar DEM, soil and land use, using five different precipitation sources. Five SWAT models were calibrated and validated to assess the relative performance of the different precipitation sources. In addition, point measurements of gridded precipitation inside the watershed were compared using GHCN-D as a reference to evaluate how well gridded datasets captured precipitation, compared to GHCN-D. Standard statistical measures revealed that DAYMET and PRISM precipitation matched well with GHCN-D weather station records; PRISM and DAYMET also most closely matched precipitation indices for GHCN-D.

The application of calibrated parameter selection and best fit techniques showed different model parameterization conditional on the precipitation sources. These parameter uncertainties can cause prediction uncertainty, mainly by simulating different water balance outputs, which shows the importance of addressing parameter uncertainty in hydrological modeling. All the calibrated models developed here have acceptable *P*-factors and *R*-factors. In addition, except for GLDAS, all calibrated streamflows are statistically similar. This study revealed the importance of precipitation source in hydrological modeling and similar thorough precipitation analysis is recommended before every hydrological application (Figures 4 and 5).

Long-term SWAT flow simulation implies that DAYMET, PRISM, and NLDAS SWAT models provided similar output to GHCN-D SWAT at both monthly and annual scales, however, GHCN-D SWAT outperformed models using PRISM and DAYMET when stations were densely located and had nearly full data coverage. In all conditions, the GHCN-D SWAT model represented the temporal pattern and variability of streamflow very well. PRISM SWAT performed better during dry flow periods, and DAYMET and NLDAS SWAT models performed best during high flow years. It can be concluded that gridded precipitation from various sources can be combined with real-time data as a hybrid data source for better hydrologic modeling. Gridded precipitation can also be used as an alternative precipitation source, especially in areas with less representation from GHCN-D and the model can significantly improve its representation of hydrologic processes with repeated iterations of calibration. This study reveals precipitation datasets affect both sensitive parameters and their corresponding ranges of values during calibration process. This shows why researchers need to be cautious when they utilize literature information to determine calibration parameters or use previously reported calibrated values in ungauged studies. It is important to note that a cautious approach is critical when using regional calibrated values of literature such as this study for ungauged studies since results are specific to the data sources used and study years. This is promising for modelers, as spatially explicit gridded data are almost always available at real time. Further research will investigate additional watersheds at different scales and locations and analyze data types at varied simulation timesteps.

## Supplementary Material

Supplement1

## Figures and Tables

**TABLE 1.** Basic characteristics of precipitation sources used in this study.

Characteristics	NLDAS	GLDAS	DAYMET	PRISM	GHCN-D (NCDC)
Source	NASA with collaborations (NOAA, Princeton University, University of Washington)	NASA with collaborations (NOAA, Princeton University, University of Washington)	Oak Ridge National Laboratory (developed by University of Montana/Peter Thornton)	Climate Group of Oregon State University	NOAA/NCDC
Product type	Interpolated	Interpolated	Interpolated	Interpolated	Gauged data
Interpolation method	Bilinear interpolation of NCEP- NCAR Reanalysis (Kalnay et al. 1996) adjusted for elevation using PRISM methodology, temporally disaggregated to one hour (Xia et al. 2012)	Similar to NLDAS	Geographically weighted regression (Thornton et al. 1997)	Geographic- and elevation-weighted regression, station weighting by topography, distance to coast, atmospheric factors (Daly et al. 2008)	Not applicable
Spatial resolution	0.125° × 0.125°	0.25° × 0.25°	1 × 1 km	2.5 arcmin (~4 × 4 km)	Not applicable
Spatial coverage	North America (U.S., southern Canada and northern Mexico)	Global	North America	The conterminous U.S. Global	
Temporal resolution	Hourly and monthly	Three-hourly and monthly	Daily	Daily, monthly	Daily
Temporal coverage	1979-present	2000-present	1980-present	1981-present	1880 to present (not in all stations)

Notes: NLDAS, North American Land Data Assimilation System; GLDAS, Global Land Data Assimilation System; DAYMET, Daily Surface Weather and Climatological Summaries; and PRISM, Parameter-elevation Regressions on Independent Slopes Model; NASA, National Aeronautics and Space Administration; NOAA, National Oceanic and Atmospheric Administration; NCEP, National Center for Environmental Prediction; NCAR, National Center for Atmospheric Research.

**TABLE 2.** List of GHCN-D Stations used in analysis with geographic location, elevation, and data description.

GHCN-D ID	Name	Longitude	Latitude	Elevation (m)	Data coverage %	Long time daily mean in mm (years used)	≥95th percentile values of annual rain days
USC00142388	Effingham	−95.3966	39.53	350.5	95.8	2.530 (1960–2016)	16.764
USC00143138	Goff 3 WSW	−95.9807	39.654	425.5	94.3	2.685 (1982–2016)	17.018
USC00143634	Hiawatha 9 ESE	−95.532	39.8356	330.4	90.7	2.505 (1960–2016)	16.002
USC00143759	Holton 7 SE	−95.7551	39.4578	320.6	96.9	2.562 (1960–2016)	16.002
USC00143810	Horton	−95.5199	39.6678	313.9	99.1	2.559 (1960–2016)	16.256
USC00146333	Perry Lake	−95.4101	39.1174	292.3	98.2	2.664 (1967–2016)	17.272
USC00148341	Valley Falls	−95.4862	39.3033	321.9	93.3	2.609 (1960–2016)	17.018

**TABLE 3.** Description of precipitation indices used in this study.

Description	Units
Very wet days (≥95th percentile)	Days
Heavy precipitation days (≥10 mm)	Days
Number of dry days (annual)	Days
Number of wet days (annual)	Days
Annual maximum number of consecutive dry days (days when precipitation < 1 mm)	Days
Annual maximum number of consecutive wet days (days when precipitation ≥ 1 mm)	Days

Calculated based on 30 years precipitation data.

**TABLE 4.** Parameters used in sensitivity analysis and their detail descriptions (Neitsch et al. 2011).

Parameter	Description	SWAT governing process	SWAT parametrized scale
CN2	SCS runoff curve number for moisture condition II	Runoff	HRU
NDTARGR	Number of days to reach target storage from current reservoir storage	Reservoir flow	Reservoir
ESCO	Soil evaporation compensation coefficient	ET	HRU
SURLAG	Surface runoff lag coefficient	Runoff	Basin
SMFMN	Melt factor for snow on December 21 (mm H_2_O/day — °C)	Snow	Basin
EPCO	Plant uptake compensation factor	ET	HRU
SFTMP	Mean air temperature at which precipitation is equally likely to be rain as snow/freezing rain (°C)	Snow	Basin
SMTMP	Threshold temperature for snowmelt (°C)	Snow	Basin
SMFMX	Melt factor on June 21 (mm H_2_O/day — °C)	Snow	Basin
RCHRG_DP	Aquifer percolation coefficient	Groundwater	HRU
GW_DELAY	Delay time for aquifer recharge (days)	Groundwater	HRU
ALPHA_BF	Baseflow recession constant	Baseflow	HRU
REVAPMN	Threshold water level in the shallow aquifer for revap (mm H_2_O)	Groundwater	HRU
OV_N	Manning’s “*n*” value for overland flow	Runoff	HRU
CH_K2	Effective hydraulic conductivity of channel (mm/h)	Channel routing	Subwatershed
TIMP	Snow temperature lag factor	Snow	Basin
GW_REVAP	Revap coefficient	Groundwater	HRU
CH_N2	Manning’s “*n*” value for the main channel	Channel routing	Subwatershed
SOL_AWC	Soil available water capacity	Runoff	HRU
GWQMN	Threshold water level in shallow aquifer for baseflow (mm H_2_O)	Groundwater	HRU
ALPHA_BNK	Bank flow recession constant or constant of proportionality	Channel routing	Subwatershed

Note: HRU, hydrologic response unit.

**TABLE 5.** SWAT Parameter definition for the most sensitive (*p* ≤ 0.05) parameters and their “best” values after first calibration iteration of SWAT models with different precipitation sources.

Parameter	Description	Parameter range	Default value	GHCN-D	DAYMET	NLDAS	GLDAS	PRISM
Alpha_BNK	Bank flow recession constant	0 to 1	0	0.76	0.76	0.83	0.36	0.47
CH_K2	Main channel saturated hydraulic conductivity (mm/h)	0 to 150	0	50.25	50.25	17.85	ns	47.25
CN2	Moisture condition II curve number	−0.2^1^ to 0.2^1^	Varies from 38 to 98	−0.08	−0.08	−0.01	−0.04	−0.06
NDTARGR	Number of days to reach target storage from current reservoir storage	0 to 200	1	40.60	40.60	ns	ns	39.4
SURLAG	Surface runoff lag time (days)	0.05 to 10	4	3.14	3.14	1.71	7.18	7.2
ESCO	Soil evaporation compensation factor	0 to 1	1.0	ns	ns	0.21	ns	ns

Note: ns is “not sensitive” at *p* ≤ 0.05.

Relative change to parameter values.

**TABLE 6.** Summary statistics for evaluation of monthly and annual calibration (2001–2013) and validation (1988–2000) of SWAT models with different precipitation sources.

	GHCN-D		DAYMET		NLDAS		GLDAS		PRISM	
Criteria	Monthly	Annual	Monthly	Annual	Monthly	Annual	Monthly	Annual	Monthly	Annual
Delaware River near Muscotah calibration (validation)										
Coefficient of determination (*R*^2^)	0.85 (0.92)	0.94 (0.96)	0.79 (0.90)	0.86 (0.95)	0.66 (0.85)	0.83 (0.96)	0.26	0.39	0.81 (0.89)	0.84 (0.97)
Nash and Sutcliffe coefficient (NSE)	0.84 (0.87)	0.87 (0.88)	0.78 (0.86)	0.79 (0.88)	0.66 (0.84)	0.82 (0.93)	0.20	0.38	0.81 (0.80)	0.82 (0.82)
Kling-Gupta efficiency (KGE)	0.78 (0.73)	0.80 (0.70)	0.75 (0.74)	0.75 (0.71)	0.73 (0.79)	0.86 (0.88)	0.45	0.44	0.80 (0.62)	0.77 (0.64)
RMSE-observations standard deviation ratio (RSR)	0.40 (0.36)	0.34 (0.33)	0.47 (0.38)	0.44 (0.34)	0.58 (0.40)	0.40 (0.25)	0.89	0.75	0.44 (0.43)	0.41 (0.40)
*P*-factor	0.91		0.92		0.81		0.78		0.89	
*R*-factor	1.89		1.95		1.96		1.53		1.96	
Delaware River at Perry Lake calibration (validation)										
*R*^2^	0.60 (0.58)	0.91 (0.95)	0.62 (0.60)	0.87 (0.98)	0.55 (0.38)	0.88 (0.94)	0.29	0.66	0.59 (0.61)	0.90 (0.98)
NSE	0.59 (0.54)	0.85 (0.84)	0.60 (0.56)	0.83 (0.88)	0.54 (0.36)	0.88 (0.94)	0.27	0.55	0.58 (0.53)	0.86 (0.80)
KGE	0.61 (0.51)	0.71 (0.65)	0.61 (0.51)	0.75 (0.67)	0.66 (0.55)	0.87 (0.95)	0.34	0.51	0.62 (0.44)	0.74 (0.61)
RSR	0.64 (0.67)	0.37 (0.38)	0.63 (0.66)	0.40 (0.34)	0.67 (0.8)	0.34 (0.24)	0.85	0.64	0.65 (0.68)	0.37 (0.43)
*P*-factor	0.88		0.90		0.79		0.82		0.87	
*R*-factor	1.86		1.94		1.97		1.53		1.99	
Reservoir outflow calibration (validation)										
*R*^2^	0.60 (0.56)	0.89 (0.95)	0.62 (0.58)	0.87 (0.98)	0.51 (0.36)	0.87 (0.94)	0.26	0.59	0.60 (0.60)	0.88 (0.98)
NSE	0.59 (0.52)	0.82 (0.82)	0.60 (0.53)	0.81 (0.86)	0.51 (0.35)	0.86 (0.94)	0.24	0.48	0.59 (0.51)	0.85 (0.76)
KGE	0.61 (0.48)	0.69 (0.62)	0.60 (0.48)	0.74 (0.65)	0.65 (0.52)	0.84 (0.96)	0.32	0.51	0.62 (0.41)	0.76 (0.57)
RSR	0.64 (0.69)	0.40 (0.41)	0.63 (0.68)	0.42 (0.36)	0.70 (0.81)	0.35 (0.24)	0.87	0.68	0.64 (0.70)	0.36 (0.47)
*P*-factor	0.85		0.87		0.76		0.77		0.87	
*R*-factor	1.86		1.95		2.00		1.51		1.98	

Notes: RMSE, root mean square error; RSR, RMSE-observations standard deviation ratio.

The model with GLDAS was not validated due to unavailable data.

**TABLE 7.** Statistics measures for evaluation of low and high flow periods of SWAT models with different precipitation sources.

Criteria	GHCN-D	PRISM	DAYMET	NLDAS	GLDAS
Dry years (flow < 90% of long-term average flow)					
*R*^2^	0.73	0.56	0.65	0.50	0.17
NSE	−0.02	0.44	−0.72	0.24	−2.06
KGE	0.43	0.68	0.23	0.58	−0.04
RSR	0.97	0.72	1.26	0.84	1.62
Wet years (flow > 110% of long-term average flow)					
*R*^2^	0.75	0.63	0.79	0.74	0.04
NSE	0.67	0.29	0.71	0.74	−2.4
KGE	0.70	0.56	0.67	0.83	0.11
RSR	0.54	0.80	0.52	0.48	1.65

The model with GLDAS was not validated due to unavailable data.

## Research Impact Statement:

Gridded precipitation datasets vary in capturing different extreme events, both dry and wet, and different precipitation data sources usage led to varying parameter calibrations in watershed modeling.
