# Supplementary material for: Comparison and Evaluation of Gridded Precipitation Datasets in a Kansas Agricultural Watershed Using SWAT
Source: J Am Water Resour Assoc. Author manuscript; Available in PMC 2021 May 16. (PMC7788048; doi:10.1111/1752-1688.12819)
Supplement: Supplement1 [file NIHMS1607524-supplement-Supplement1.docx]

# **Supplementary**

**S 1: HMS Workflow**

The workflow and underlying HMS infrastructure were developed by EPA’s Office of Research and Development (ORD) with the objective of developing a collection of interoperable and reusable water quantity and quality data provisioning and modeling components. All HMS components are available in two forms: RESTful web services and a desktop library. The components can be used to compose problem specific workflow. A simple work flow for this study was developed using HMS data provisioning and geo-processing components. The workflow was composed of automated tasks such as downloading precipitation from source respective websites, shifting time-series to local time zone if required, computing daily precipitation from less than daily temporal resolution if required, flagging missing values, and preparing SWAT consumable precipitation data files.

HMS data provisioning components used in the workflow access the respective web services of the data providers. GLDAS precipitation web service from NASA requires latitude, longitude, start date and time, and end date and time. NLDAS precipitation data web service requires NLDAS grid coordinates, start date and time, and end date and time. HMS components for NLDAS precipitation data takes latitude and longitude as arguments and converts them into NLDAS coordinates required by NASA’s NLDAS web service. NASA’s NLDAS and GLDAS web services provide precipitation data in Greenwich Mean Time (GMT). HMS NLDAS and GLDAS precipitation data-provisioning components adjust the precipitation time series from GMT to local time using latitude and longitude. As mentioned previously, NLDAS and GLDAS precipitation data from NASA web services are listed as hourly and three-hour time series. HMS NLDAS and GLDAS precipitation data provisioning components have the option to calculate daily precipitation time series from hourly and three-hour time series, as used in this workflow.

DAYMET and PRISM data were available from the web services of their providers as daily time series in local time. Therefore, HMS DAYMET and PRISM data provisioning components do not have to perform any temporal and spatial processing to provide daily time series in local time. PRISM data is hosted by Oregon State University (OSU) and dispensed as a layer containing all data rods within user-specified spatial extents, making data download and extraction quite slow. HMS PRISM precipitation data provisioning component calls PRISM data web services hosted by Colorado State University (CSU) at <https://alm.engr.colostate.edu/cb/issue/30489> for downloading precipitation data as a data rod for a user-specified location.

HMS GHCN-D precipitation data provisioning component downloads data from GHCN-D web service. Data from the GHCN-D web service is for a user-specified weather station. HMS GHCN-D data provisioning component calls another GHCN-D web service to determine weather stations within the user-specified area of interest (AOI). HMS GHCN-D precipitation data provisioning component calculates daily precipitation time series from the GHCN-D provided temporal resolution which varies from one station to another. HMS GHCN-D precipitation data provisioning component also flags missing data in the time series.

Table S1: Sensitivity indices for SWAT parameters with different precipitation settings. Highly sensitivity parameters (at p= 0.05) are highlighted.

| Parameter Name |  | GHCN-D SWAT | | | DAYMET SWAT | | | NLDAS SWAT | | | GLDAS SWAT | | | PRISM SWAT | | |
| --- | --- | --- | --- | --- | --- | --- | --- | --- | --- | --- | --- | --- | --- | --- | --- | --- |
|  |  | t-Stat | p-Value | Rank | t-Stat | p-value | Rank | t-Stat | p-Value | Rank | t-Stat | p-Value | Rank | t-Stat | p-Value | Rank |
| CN2.mgt |  | -13.86 | 0.00 | 1 | -12.81 | 0.00 | 1 | -19.61 | 0.00 | 1 | -11.13 | 0.00 | 1 | -16.34 | 0.00 | 1 |
| ALPHA_BNK.rte |  | 3.60 | 0.00 | 2 | 3.11 | 0.00 | 3 | 2.62 | 0.01 | 4 | 2.22 | 0.03 | 3 | 3.00 | 0.00 | 3 |
| SURLAG.bsn |  | 3.38 | 0.00 | 3 | 3.32 | 0.00 | 2 | 3.53 | 0.00 | 3 | 3.51 | 0.00 | 2 | 3.53 | 0.00 | 2 |
| NDTARGR.res |  | -2.68 | 0.01 | 4 | -2.24 | 0.03 | 4 | -1.72 | 0.09 | 7 | -0.34 | 0.73 | 17 | -2.30 | 0.02 | 5 |
| CH_K2.rte |  | -2.42 | 0.02 | 5 | -2.13 | 0.03 | 5 | -2.37 | 0.02 | 5 | -1.46 | 0.15 | 4 | -2.51 | 0.01 | 4 |
| ESCO.hru |  | 1.66 | 0.10 | 6 | 1.28 | 0.20 | 7 | -4.33 | 0.00 | 2 | 0.27 | 0.79 | 20 | -0.94 | 0.35 | 11 |
| TIMP.bsn |  | 1.41 | 0.16 | 7 | 1.17 | 0.24 | 8 | 1.32 | 0.19 | 9 | 0.67 | 0.50 | 11 | 1.24 | 0.22 | 6 |
| REVAPMN.gw |  | -1.23 | 0.22 | 8 | -1.32 | 0.19 | 6 | -1.21 | 0.23 | 11 | -1.29 | 0.20 | 6 | -1.23 | 0.22 | 7 |
| OV_N.hru |  | 1.12 | 0.26 | 9 | 1.06 | 0.29 | 10 | 1.27 | 0.20 | 10 | 1.23 | 0.22 | 7 | 1.19 | 0.23 | 9 |
| GW_REVAP.gw |  | 1.05 | 0.29 | 10 | 0.97 | 0.33 | 11 | 1.12 | 0.26 | 12 | 0.91 | 0.36 | 8 | 1.22 | 0.22 | 8 |
| SOL_AWC(..).sol |  | -0.84 | 0.40 | 11 | -0.77 | 0.44 | 12 | 0.20 | 0.84 | 19 | -0.40 | 0.69 | 15 | -0.39 | 0.70 | 16 |
| SMTMP.bsn |  | -0.74 | 0.46 | 12 | -1.13 | 0.26 | 9 | -1.44 | 0.15 | 8 | -0.44 | 0.66 | 14 | -0.87 | 0.38 | 12 |
| GW_DELAY.gw |  | -0.59 | 0.55 | 13 | -0.59 | 0.56 | 16 | -0.34 | 0.73 | 15 | -0.03 | 0.98 | 21 | -0.44 | 0.66 | 15 |
| RCHRG_DP.gw |  | 0.58 | 0.56 | 14 | 0.68 | 0.49 | 14 | 0.31 | 0.76 | 16 | 1.39 | 0.16 | 5 | 0.47 | 0.64 | 14 |
| EPCO.hru |  | 0.53 | 0.60 | 15 | 0.66 | 0.51 | 15 | 1.89 | 0.06 | 6 | 0.62 | 0.54 | 12 | 1.12 | 0.26 | 10 |
| SMFMN.bsn |  | -0.42 | 0.68 | 16 | -0.71 | 0.48 | 13 | -0.87 | 0.38 | 13 | -0.81 | 0.42 | 9 | -0.65 | 0.52 | 13 |
| SFTMP.bsn |  | -0.40 | 0.69 | 17 | -0.55 | 0.59 | 17 | -0.47 | 0.64 | 14 | -0.34 | 0.73 | 16 | -0.33 | 0.74 | 18 |
| GWQMN.gw |  | -0.39 | 0.70 | 18 | -0.20 | 0.84 | 19 | -0.25 | 0.80 | 18 | -0.58 | 0.56 | 13 | -0.36 | 0.72 | 17 |
| ALPHA_BF.gw |  | -0.29 | 0.77 | 19 | -0.24 | 0.81 | 18 | -0.09 | 0.92 | 20 | -0.30 | 0.76 | 18 | -0.17 | 0.87 | 20 |
| SMFMX.bsn |  | 0.20 | 0.84 | 20 | 0.12 | 0.90 | 20 | 0.30 | 0.77 | 17 | 0.28 | 0.78 | 19 | 0.27 | 0.79 | 19 |
| CH_N2.rte |  | 0.04 | 0.96 | 21 | 0.09 | 0.93 | 21 | 0.00 | 1.00 | 21 | 0.69 | 0.49 | 10 | -0.02 | 0.98 | 21 |

** Detail parameter descriptions are given in Table 4. The t-stat is the coefficient of the parameter divided by its standard error and used to measure the precision with which the regression coefficient is measured (Abbaspour 2015); the larger the t-stat (absolute value), the more sensitive the parameter. The p-value measures how significant that parameter is to the sensitivity analysis. Ranking parameters by significance enhances model understanding and identifies the most important controls of model behavior. Rank is the rank based on absolute t-Stat rank.

Table S2: Tukey multiple comparisons of means at 95% family-wise confidence level during calibration and validation periods.

| Precipitation pair | difference between means of the two groups | the lower end point of the confidence interval at 95% | the upper end point of the confidence interval at 95% | p-value after adjustment for the multiple comparisons | difference between means of the two groups | the lower end point of the confidence interval at 95% | the upper end point of the confidence interval at 95% | p-value after adjustment for the multiple comparisons |
| --- | --- | --- | --- | --- | --- | --- | --- | --- |
| GHCN-D-DAYMET SWAT | 0.02 | -0.07 | 0.10 | 0.99 | -0.04 | -0.09 | 0.01 | 0.18 |
| GLDAS-DAYMET SWAT | -0.11 | -0.20 | -0.03 | 0.00 |  |  |  |  |
| NLDAS-DAYMET SWAT | -0.06 | -0.14 | 0.03 | 0.36 | -0.02 | -0.07 | 0.04 | 0.82 |
| PRISM-DAYMET SWAT | 0.02 | -0.06 | 0.11 | 0.94 | -0.04 | -0.10 | 0.01 | 0.14 |
| GLDAS-GHCN-D SWAT | -0.13 | -0.21 | -0.05 | 0.00 |  |  |  |  |
| NLDAS-GHCN-D SWAT | -0.07 | -0.15 | 0.01 | 0.14 | 0.02 | -0.03 | 0.08 | 0.63 |
| PRISM-GHCN-D SWAT | 0.01 | -0.07 | 0.09 | 1.00 | 0.00 | -0.06 | 0.05 | 1.00 |
| NLDAS-GLDAS SWAT | 0.06 | -0.02 | 0.14 | 0.31 |  |  |  |  |
| PRISM-GLDAS SWAT | 0.14 | 0.05 | 0.22 | 0.00 |  |  |  |  |
| PRISM-NLDAS SWAT | 0.08 | 0.00 | 0.16 | 0.07 | -0.03 | -0.08 | 0.03 | 0.56 |

**Tukey multiple pairwise-comparisons identified differences between performances of each precipitation dataset. The difference in the group means (provided in the table for both periods as the difference between means of the two groups), the lower end point of the interval (the lower end point of the confidence interval at 95%), the upper end point (the upper end point of the confidence interval at 95%) and the p-value after adjustment for the multiple comparisons are given in the table.

Table S3: Summary results of model evaluation with five different precipitation sources. The value 1denotes the conditions where statistical criteria were met and 0 were not.

| Sites | Stat. | GHCN-D SWAT | | | | | DAYMET SWAT | | | | | NLDAS SWAT | | | | | GLDAS SWAT* | | | PRISM SWAT | | | | |
| --- | --- | --- | --- | --- | --- | --- | --- | --- | --- | --- | --- | --- | --- | --- | --- | --- | --- | --- | --- | --- | --- | --- | --- | --- |
|  |  | Monthly | | Annually | | Total | Monthly | | Annually | | Total | Monthly | | Annually | |  | Monthly | Annually | Total | Monthly | | Annually | | Total |
|  |  | Cal | Val | Cal | Val |  | Cal | Val | Cal | Val |  | Cal | Val | Cal | Val | Total | Cal | Cal |  | Cal | Val | Cal | Val |  |
| **Delaware River near Muscotah** | R2 | 1 | 1 | 1 | 1 | 4 | 1 | 1 | 1 | 1 | 4 | 0 | 1 | 1 | 1 | 3 | 0 | 0 | 0 | 1 | 1 | 1 | 1 | 4 |
|  | NSE | 1 | 1 | 1 | 1 | 4 | 1 | 1 | 1 | 1 | 4 | 1 | 1 | 1 | 1 | 4 | 0 | 0 | 0 | 1 | 1 | 1 | 1 | 4 |
|  | KGE | 1 | 1 | 1 | 1 | 4 | 1 | 1 | 1 | 1 | 4 | 1 | 1 | 1 | 1 | 4 | 0 | 0 | 0 | 1 | 1 | 1 | 1 | 4 |
|  | RSR | 1 | 1 | 1 | 1 | 4 | 1 | 1 | 1 | 1 | 4 | 1 | 1 | 1 | 1 | 4 | 0 | 0 | 0 | 1 | 1 | 1 | 1 | 4 |
| **Delaware River at Perry Lake** | R2 | 0 | 0 | 1 | 1 | 2 | 0 | 0 | 1 | 1 | 2 | 0 | 0 | 1 | 1 | 2 | 0 | 0 | 0 | 0 | 0 | 1 | 1 | 2 |
|  | NSE | 1 | 0 | 1 | 1 | 3 | 1 | 1 | 1 | 1 | 4 | 0 | 0 | 1 | 1 | 2 | 0 | 0 | 0 | 1 | 0 | 1 | 1 | 3 |
|  | KGE | 1 | 1 | 1 | 1 | 4 | 1 | 1 | 1 | 1 | 4 | 1 | 1 | 1 | 1 | 4 | 0 | 0 | 0 | 1 | 0 | 1 | 1 | 3 |
|  | RSR | 0 | 0 | 1 | 1 | 2 | 0 | 0 | 1 | 1 | 2 | 0 | 0 | 1 | 1 | 2 | 0 | 0 | 0 | 0 | 0 | 1 | 1 | 2 |
| **Reservoir outflow** | R2 | 0 | 0 | 1 | 1 | 2 | 0 | 0 | 1 | 1 | 2 | 0 | 0 | 1 | 1 | 2 | 0 | 0 | 0 | 0 | 0 | 1 | 1 | 2 |
|  | NSE | 1 | 0 | 1 | 1 | 3 | 1 | 0 | 1 | 1 | 3 | 0 | 0 | 1 | 1 | 2 | 0 | 0 | 0 | 1 | 0 | 1 | 1 | 3 |
|  | KGE | 1 | 0 | 1 | 1 | 3 | 1 | 0 | 1 | 1 | 3 | 1 | 1 | 1 | 1 | 4 | 0 | 0 | 0 | 1 | 0 | 1 | 0 | 2 |
|  | RSR | 0 | 0 | 1 | 1 | 2 | 0 | 0 | 1 | 1 | 2 | 0 | 0 | 1 | 1 | 2 | 0 | 0 | 0 | 0 | 0 | 1 | 1 | 2 |
| Total Month | | 13 | | | | | 14 | | | | | 11 | | | | | 0 | | | 12 | | | | |
| Total Annual | | 24 | | | | | 24 | | | | | 24 | | | | | 0 | | | 23 | | | | |
| Total | | 37 | | | | | 38 | | | | | 35 | | | | | 0 | | | 35 | | | | |

**Summary results of model evaluation (Nash-Sutcliffe Efficiency [NSE], coefficient of determination [R2], and Kling-Gupta Efficiency [KGE] and RMSE-observations standard deviation ratio [RSR]) with five different precipitation sources for all three sites (Delaware River near Muscotah, Delaware River at Perry Lake and Reservoir outflow). For stream flow NSE values >0.50 and R2 >0.60 considered to be a satisfactory level for monthly scales. A KGE value >0.50 and RSR value <0.60 are considered satisfactory. In addition, NSE values >0.65, R2 >0.80, KGE values >0.60, and RSR value <0.50 are considered satisfactory at an annual scale in this study. The value 1denotes the conditions where statistical criteria were met and 0 were not.

* The model with GLDAS was not validated due to unavailable data
